# Postural Correlates of Pollution Perception

**DOI:** 10.3390/brainsci12070869

**Published:** 2022-06-30

**Authors:** Mbarka Akounach, Thierry Lelard, Anaïs Beaumont, Sylvie Granon, Harold Mouras

**Affiliations:** 1 UR-UPJV 4559, Functional Neurosciences Laboratory, Health Research Universitary Center, Medecine UFR, Picardy Jules Verne University, CEDEX, 80054 Amiens, France; mbarkaakounach@gmail.com (M.A.); anaisbeaumont@orange.fr (A.B.); 2 NeuroPSI—Paris-Saclay Institute of Neuroscience, Team Neurobiology of Decision-Making, 91400 Saclay, France; sylvie.granon@universite-paris-saclay.fr; 3 UR-UPJV 3300, Physiological Adaptations to Exercise and Exercise Rehabilitation, Sport Sciences UFR, Picardy Jules Verne University, 80025 Amiens, France; thierry.lelard@u-picardie.fr

**Keywords:** posturography, mental simulation, affective neuroscience, pollution

## Abstract

In our contemporary societies, environmental issues are more and more important. An increasing number of studies explore the biological processes involved in environment perception and in particular try to highlight the mechanisms underlying the perception of environmental scenes by our brain. The main objective of the present study was to establish whether the visualization of clean and polluted environmental scenes would lead to differential postural reactions. Our hypothesis was based on a differential postural modulation that could be recorded when the subject is confronted with images representing a “polluted” environment, differential modulation which has been reported in previous studies in response to painful-scenes compared to non-painful scenes visualization.Thirty-one subjects participated in this study. Physiological measurements [heart rate variability (HRV) and electrodermal activity] and postural responses (Center Of Pression—COP—displacements) were recorded in response to perception of polluted or clean environmental scenes. We show, for the first time, that images representing polluted scenes evoke a weaker approach movement than images representing clean scenes. The displacement of the COP in the anteroposterior axis reflects an avoidance when subjects visualize “polluted” scenes. Our results demonstrate a clear distinction between “clean” and “polluted” environments according to the postural change they induce, correlated with the ratings of pleasure and approach evoked by images.

## 1. Introduction

Interaction with the environment involves a fine-grained articulation between sensory and motor processes influenced in particular by the sensory modalities and emotions involved [1]. Studies on the description of behaviors and attitudes associated with emotions have built on the pioneering work of Darwin and William James [2]. Regarding the link between emotional and motor processes, current neuroscientific theories propose that during the emotional information processing chain motor processes are activated in parallel in an almost automatic way [3].

Different physiological responses have been studied in response to the processing of emotional stimuli such as pupillary dilation (reflecting sympathetic nervous system activity; [4]) or, in a large majority of studies, facial expression [5]. Recently, a new research direction has emerged with the recording of postural control during the processing of emotional stimuli [6,7]. Experimentally, a number of studies have shown approach or avoidance behavior in response to pleasant or unpleasant stimuli, respectively [8]. Sometimes motor responses appear more complex, such as motor sideration (freezing) in response to aversive stimuli [9].

Over the past decade, the empathy for pain model has been widely used to study responses (central, peripheral, psychological) to the processing of socioemotional stimuli. In particular, a series of studies conducted by our team have used this model to record associated postural modulations [10,11,12]. A significant effect was first demonstrated [10] for postural changes associated with painful and non-painful situations. The embodied theory of emotion indicates that the emotional response is closely related to the subject’s involvement in the situation. This theory is based on imagining ourselves in a situation already experienced by us or by another subject by simulating actions and perception involving an important mechanism that activates our motor models and is considered as an important characteristic in terms of adaptive behavioral skills that allow the subject to anticipate the outcome of a movement. This process allows us to understand the behavior of others and helps to facilitate interactions with the environment. This dimension was taken into consideration in a subsequent study [11] to test the effects of mental simulation in painful and non-painful situations. Thus, two experimental conditions were compared, a so-called “passive” one without specific instructions and a “mental simulation observation” representing the embodiment or mental simulation process. These two conditions induced different posturographic results. A freezing response was observed towards visual pain stimuli, specifically in the case of active observation [11]. Indeed, automatic postural and physiological responses to visual stimulation appear to reflect an instance of embodiment. Thus, it is hypothesized that the perception and imagination of emotional scenes could reactivate associated motor responses, including responses involving postural adjustments. This mechanism can be used to understand another person’s intentions [7].

Previous studies have explored the subjective feelings of healthy human participants in environments or landscapes. The perception of landscapes involves both objective and subjective dimensions. As postulated by Meinig [13], any landscape “is composed not only of what lies before our eyes but what lies within our heads,” supporting landscape perception as an active construction of our brain. Shedding light on the emotional dimension of landscape perception, the preference for natural overt human-influenced environments has been reported in different cultures [14] and at a psychophysiological level [15,16]. In a previous study [17], we explored the subjective feelings (displeasure/pleasure and approach/avoidance) recorded in response to polluted and clean scenes. This research sheds light on the importance of the emotional processing of polluted environmental scenes to construct a global visual experience of pollution and to guide relevant behavior toward these scenes. In a similar vein, within the field of postural correlates of socioemotional information processing, research has shown the modulatory effect of mental simulation on the postural correlates of empathy for pain [11,12]. In our present research, we tested this effect in the context of environmental scenes and pollution to understand how this effect could be identified as an important lever to change decision-making processes linked to pollution.

## 2. Methods

### 2.1. Subjects

Thirty-one volunteers (14 males and 17 females, mean age 25 ± 6.4 years old) with no known visual or motor impairment and no previous or current treatment for psychiatric or neurological disorders were included in this study. All participants signed an informed consent form. Experimental procedures were conducted in accordance with the ethical standards of the Declaration of Helsinki and were approved by the local ethics committee (CER Université Paris Saclay, Orsay, France).

### 2.2. Stimuli

Twenty-two visual stimuli representing polluted environmental scenes (eleven images) or clean environmental scenes (eleven images) for each condition (active viewing, passive viewing) were extracted from a recently published database [17]. Scenes evoking average pleasure as well as average approach desire statistically greater than 6 were chosen for the “clean” condition, and those evoking average pleasure as well as average approach desire statistically less than 4 were chosen for the “polluted” condition. The presentation of the stimuli was controlled by a computer running E-Prime 2 software (Psychology Software Tools, Inc., Pittsburgh, PA, USA).

### 2.3. Procedure

**Figure 1:**
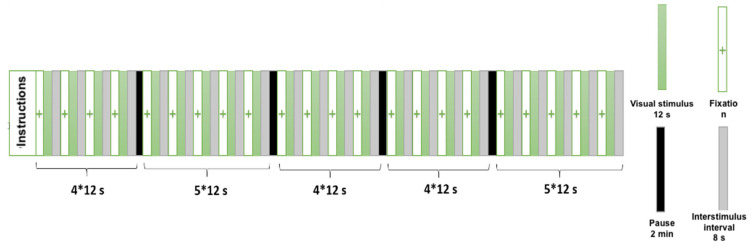
Temporal course of experimental session.

Initially, participants were asked to place the Empatica E4 watch on their dominant wrists before the start of the experiment, as the coupling of the electrodes with the skin can take about 10–15 min. Participants stood barefoot in the middle of the force platform (AMTI BP600 × 400, Watertown, MA, USA) and were asked to maintain a comfortable bipedal position with their arms relaxed at their sides and their feet pointing 30° outward. Visual stimuli were then presented to the participants on a screen placed 1 m 30 away from the subject’s eyes and directly connected to the computer presenting the stimuli. The presentation of the images and the time course of the experimental session are illustrated in Figure 1. During a first recording session (corresponding to the “passive observation condition”), participants were only instructed to look at the presented images and “remain as still as possible, do not make any voluntary movements”. In this first session, the 22 images (11 images from the “clean” condition and 11 images from the “polluted” condition) were presented randomly. Each trial started with a fixation cross for 2 s, continued with the presentation of the stimulus for a duration of 12 s, and then was followed by an inter-stimulus interval of 10 s in order to allow a return to a basal state of the different physiological indices (electrodermal response, heart rate). After the presentation of four stimuli, participants were asked to move their legs and sit comfortably to avoid fatigue for 2 min.

During a second session (corresponding to the “mental simulation observation” condition), participants were instructed to imagine themselves in the presented situation. Additional stimuli (11 images from the “clean” condition and 11 images from the “polluted” condition) were presented randomly while participants maintained the same comfortable bipedal position with the instruction: “Your task is now to look at the images that are about to be presented by imagining yourself as an actor in the situation.”

Participants’ subjective reactions were collected on the same dimensions as those tested for the original image base [17]. The forty-four visual stimuli of the two conditions (“clean” and “polluted”) were presented to the participants using E-Prime 2 software (Psychology Software Tools, Inc., Pittsburgh, PA, USA). Each stimulus was presented for 3 s and was followed by two questions, always presented in the same order, to which the participant had to respond on the computer keyboard without a time limit, but with the instruction to answer as quickly as possible. For each scene, the participant had to quantify on Likert-type scales ranging from 1 to 9 the pleasure and desire to approach evoked by the scene.

### 2.4. Data Recording and Analyses

Force and moment data were recorded using a force platform (AMTI BP600 × 400, Watertown, MA, USA) at 40 Hz. COP positions were calculated using a custom Matlab function (R2017a, The MathWorks Inc., Natick, MA, USA). The calculation of these positions was done using the equations provided within the AMTI Biomechanics Force Platform Installation Manual (page 29, Version 4.4, April 2017). For each stimulus, the COP displacement in the anteroposterior direction corresponding to the difference between the COP position during the stimulus presentation (12 s) and the COP position 500 ms before stimulus onset was calculated and subtracted to each COP-position recorded during the stimulus presentation period. Two parameters were analyzed: (a) the mean COP displacement on the anteroposterior axis; and (b) the standard deviation of the mean COP position on the anteroposterior axis.

Electrodermal activity and heart rate were recorded by a wireless watch (Empatica E4, Boston, MA, USA). The watch was placed on the participants’ non-dominant wrist (to minimize motion artifacts). The E4 wristband had to be tight enough to ensure that the electrodes for recording the electrodermal response did not change position on the skin during normal movement. The watch contained four sensors: (1) an electrode for electrodermal activity (electrodermal activity), (2) 3-axis accelerometer, (3) a temperature sensor, and (4) a photoplethysmograph to measure pulse blood volume (PBV) from which it derives heart rate HR and interbeat interval (IBI). Using Empatica software, the data were uploaded to Empatica Connect and subsequently analyzed in their raw form.

The electrodermal activity signal was processed using Ledalab, a specific open-source toolkit implemented within MATLAB for electrodermal activity processing (http://ledalab.de/ accessed on 4 April 2021). Continuous decomposition analysis was applied to separate the tonic and phasic components. Indeed, there are two major components for electrodermal activity analysis: the tonic component is a slowly changing part of the electrodermal activity signal that is mainly related to the overall arousal of a subject during a situation, while the phasic component is the rapidly changing part of the electrodermal activity signal, which occurs in relation to the single stimulus responses considered in our study.

Regarding heart rate, heart rate variability (HRV) is a commonly used measure that is derived from the data collected. It is the time interval between individual heartbeats. It is used to estimate the instantaneous heart rate. An algorithm built into the Empatica watch removes incorrect peaks due to noise in the signal. Time domain measurements are calculated by examining the segments between heartbeats or normal intervals (NN) measured in milliseconds (ms). To perform this analysis, an adaptation of an open-source Python code HRVAnalysis (https://github.com/Aura-healthcare/hrv-analysis accessed on 5 May 2021) was performed. In order to align our results with the relevant literature, an analysis of the most commonly used time domain indices was performed: RMSSD (the root mean square of successive differences), and SDNN (the standard deviation of normal inter-beat intervals (NN).

### 2.5. Questionnaires Regarding Motivation and Attachment to Nature

In order to understand the interindividual differences within physiological responses, two standardized questionnaires were used before the experiment. The first used was the *Environmental Concerns (INS;* [18]) a scale that measures the inclusion of nature in the self. It is one of seven measures used to assess the extent to which humans are connected to nature. Regarding the scores provided by this scale, participants are divided into two groups, the first group G1 represents those who have a score of 6 or 7 (strongly connected to nature) and the second group G2 represents those who have a score of less than 5 (Moderately or not at all connected to nature). Secondly, the French validated version of the *Motivation Towards the Environment Scale (MTES;* [19]), i.e., the *“Echelle de Motivation vis à vis des Comportements Ecologiques” (EMCE;* [20]), a motivation scale for ecological behaviors, consisting of 24 items representing six motivation subscales. These six subscales correspond to the different types of motivation identified by Deci and Ryan [21], i.e., intrinsic motivation, four types of extrinsic motivation (integrated regulation, identified, introjected and external) and amotivation.

### 2.6. Statistics Analysis

The data were checked for a normal distribution using the Shapiro-Wilk test. Next, we performed a two-factor ANOVA (observation type: active or passive and image type: clean or polluted).

With regard to the analyses of the data, the analysis of the scores obtained for the two dimensions pleasure and desire of approach evoked by the images, a two-factor ANOVA was performed for each type of image and observation.

In the following analysis, the average amplitude of the electrodermal response peaks during the experimental conditions was studied. The Shapiro-Wilk test was performed to test the normality assumption. Since the values were not normally distributed, a non-parametric Kruskal-Wallis test was used to compare the responses to clean and polluted landscapes. A Spearman rank correlation was used to measure the degree of association between variables. When several comparisons are made, a correction is applied (Bonferroni), to reduce the probability of committing a type I error. All analyses were performed with the SPSS 19.0 statistical package and R software. A significance level of *p* = 0.05 was used for all statistical analyses.

## 3. Results

### 3.1. Subjective Ratings

**Figure 2:**
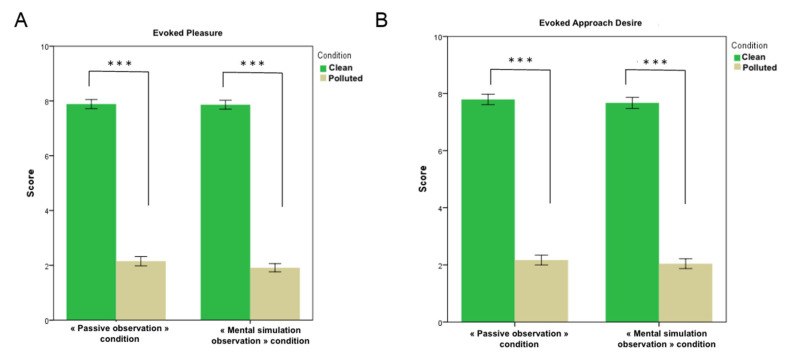
Mean Evoked Pleasure (**A**) and Evoked Approach Desire (**B**) (mean ± SEM) on 44 images (22 presenting a polluted environment and 22 presenting a clean environment) according to Passive observation condition (left side of each panel) and Mental simulation observation condition (right side of each panel). Statistical differences are shown as *** *p* < 0.001.

Figure 2 shows the subjective ratings recorded across the different experimental conditions for the “Evoked Pleasure” and “Evoked Approach Desire” dimensions. First, mean Evoked Pleasure in the Clean condition was significantly higher than in the Polluted condition (*p* = 1.80 × 10^−54^), either in the passive or mental simulation observation conditions. No significant difference was detected between the active and passive conditions (*p* = 0.624), either in the Clean or Polluted conditions. Secondly, mean Evoked Approach Desire in the Clean condition was significantly higher than in the Polluted condition (*p* = 2.24.10^−48^). No significant difference was detected between the active and passive conditions (*p* = 0.530), either in the Clean or Polluted conditions. These results confirm that scenes showing unpolluted landscapes evoke significantly greater pleasure and approach desire than those showing polluted landscapes.

### 3.2. Posturographic Data

When viewing a polluted image, the subjects tend to move back, and the value of the displacement of the COP is then negative (M = −0.334 ± 1.42 mm in passive condition, M = −0.3454 ± 1.43 mm in mental simulation condition). Clean images tend to generate approach behavior reflected by positive values of COP displacement (M = 0.38 ± 1.25 mm in passive condition and M = 0.7812 ± 1.42 mm in mental simulation condition).

**Figure 3:**
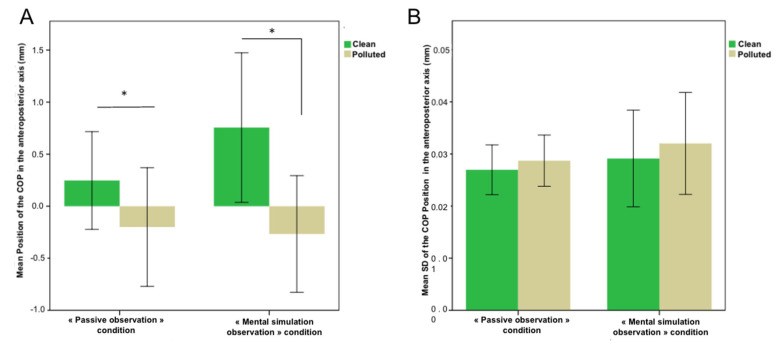
Mean Position (**A**) and Position’s Standard Deviation (**B**) of the COP along the anteroposterior axis (mm) according to Passive observation condition (left side of each panel) and Mental simulation observation condition (right side of each panel). Significant differences exist for viewing a polluted scene and viewing a clean scene (*p* = 0.011). * *p* < 0.05, means ± standard deviations.

As shown in Figure 3, for the average position of the COP in the anteroposterior axis, the difference between the “Clean” and “Dirty” conditions was significant both during passive observation (F = 0.113, *p* = 0.025, power = 0.738) and during observation under “mental simulation” conditions. On the other hand, we did not observe any difference between passive observation and active observation (*p* = 0.43) but only a non-significant trend evoking a greater approach when faced with a clean scene and a withdrawal when faced with a polluted scene during mental simulation compared to passive observation.

A Spearman correlation test showed a positive correlation between the means of COP displacement and the score given either for pleasure (r = 0.272) or for evoked approach desire (r = 0.234). The correlation is significant at the threshold *p* < 0.01 (bilateral).

### 3.3. Physiological Data

**Figure 4:**
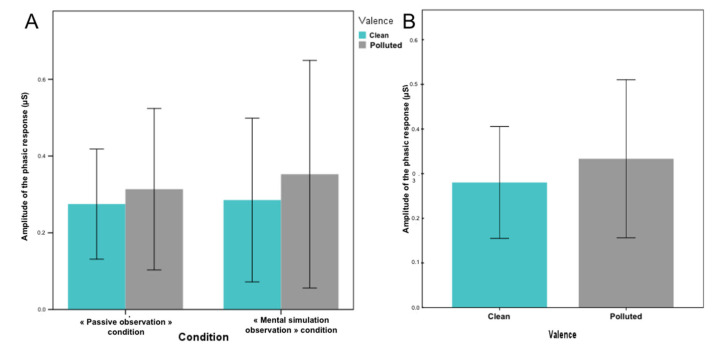
Mean amplitude of the phasic response of electrodermal activity for Clean (blue) and the Polluted (Grey) condition considering the difference between the passive and mental simulation observation conditions (**A**) or averaged on all experimental conditions (**B**).

The purpose of measuring electrodermal activity was to determine whether emotions induced by landscape visualization could induce sympathetic nervous system responses. Environmental scenes of 12 s duration should be appropriate to evoke event-related responses. Therefore, in the present study, it was expected that the electrodermal activity signal would differ across landscapes based on their emotional determinants. The measure of electrodermal activity was the maximum signal amplitude (phasic component) obtained for each stimulus relative to the baseline level (representing the baseline). A slight difference in electrodermal response was observed when subjects viewed a polluted landscape (M = 0.33 ± 0.65 µS) compared to a clean landscape (M = 0.28 ± 0.46 µS; Figure 4A). In group comparisons revealed higher amplitudes of electrodermal activity when participants viewed landscapes during mental simulation (active viewing) compared to passive viewing. Indeed, images of “Polluted” landscapes elicited a larger response (0.352390 ± 0.76 µS for active observation and 0.313493 ± 0.53 µS for passive observation) compared to “Clean” landscapes (0.285404 ± 0.55 µS for active observation and 0.274857 ± 0. 36 µS for passive observation; Figure 4B). However, no significant differences were observed between the two types of landscapes or between the two types of observation (*p* = 0.991; ddl = 2; F = 0.009).

Regarding heart rate, no significant differences were demonstrated for the different heart rate related parameters (*p* = 0.553; ddl = 1; F = 0.355). However, it is interesting to note that there is a slight increase in the response to polluted images as compared to clean ones for (i) the Root Mean Square of Successive R-R intervals (RMSSD) considered as representing parasympathetic stimulation and reflecting changes in autonomic tone that are primarily regulated by the vagus nerve [22,23]; and (ii) Standard Deviation of all NN intervals (SDNN) reflecting a high level of arousal. The group comparison revealed slightly higher responses during the observation of landscapes during the mental simulation (active observation) compared to passive observation (see Table 1).

### 3.4. Behavioral Questionnaires: Motivation and Attachment to Nature

**Figure 5:**
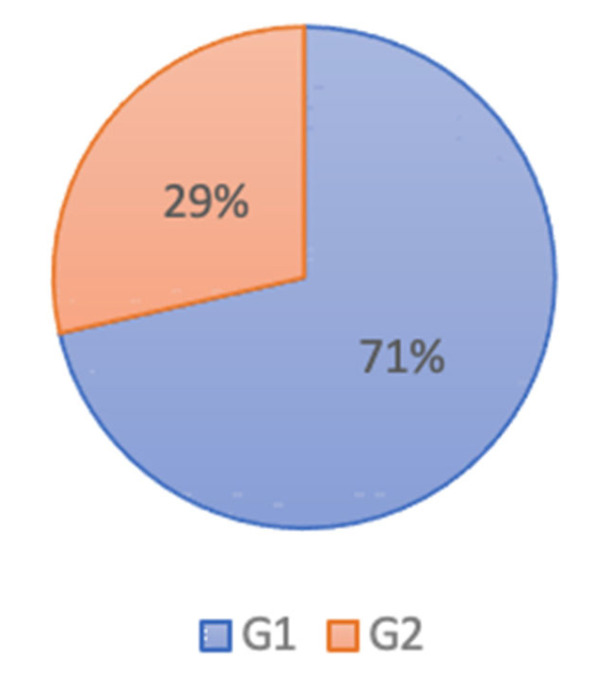
Distribution of participants by INS score; G1: score ≤ 5; G2: score < 5.

For the INS scale, as depicted on Figure 5, 71% of our subjects are strongly connected to nature, which explains the reaction of the participants expressed by their avoidance of polluted scenes. It is suggested that the more one is connected to nature the more one tends to act towards polluted landscapes.

**Figure 6:**
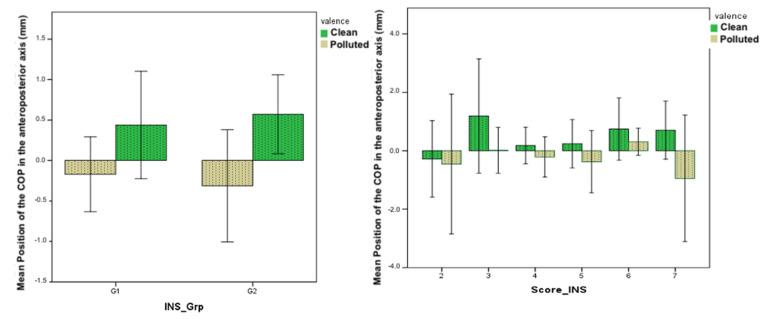
(**left**) Displacement of the COP in the AP axis according to the connection to nature. (**right**) Shift of the COP in the AP axis according to the INS score (Inclusion of Nature in the Self index).

People in the second group (Figure 6, left) tended to approach a “clean” scene and avoid a “polluted” landscape compared to the first group. The analyses showed no significant effect (*p* = 0.59). An analysis of postural responses to “polluted” or “clean” environmental scenes according to each INS scale score is presented in Figure 6 on the right side. Subjects with a strong connection to nature (those with a score of 7) tend to avoid “polluted” landscapes compared to other subjects. This effect is not significant (*p* = 0.44), as the number of subjects giving each score is limited.

**Figure 7:**
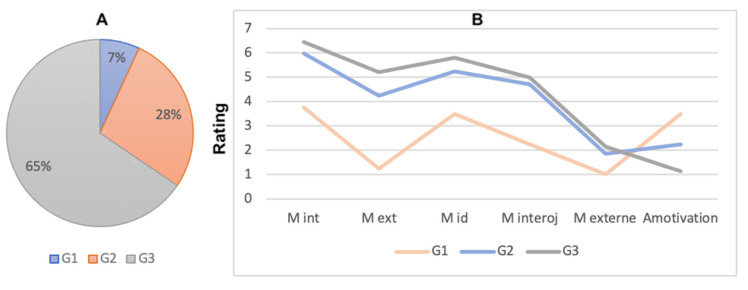
(**A**) Distribution of participants according to WES scores (G1: Low motivation; G2: Moderate motivation; G3: High motivation). (**B**) Graphical representation of average motivation scores by cluster for a four-cluster solution.

For the EMCE scale, participants are grouped into three groups based on their response (Figure 7, left side). The first group, G1, contained 7% of all cases (*n* = 2) with low scores on all types of motivation except amotivation. The second group represented 28% of participants (*n* = 8) with moderate scores on all five MTES subscales. Finally, group three, G3, was the largest, comprising 65% of subjects (*n* = 20). This group scored high on all types of motivation except amotivation (Figure 7B).

**Figure 8:**
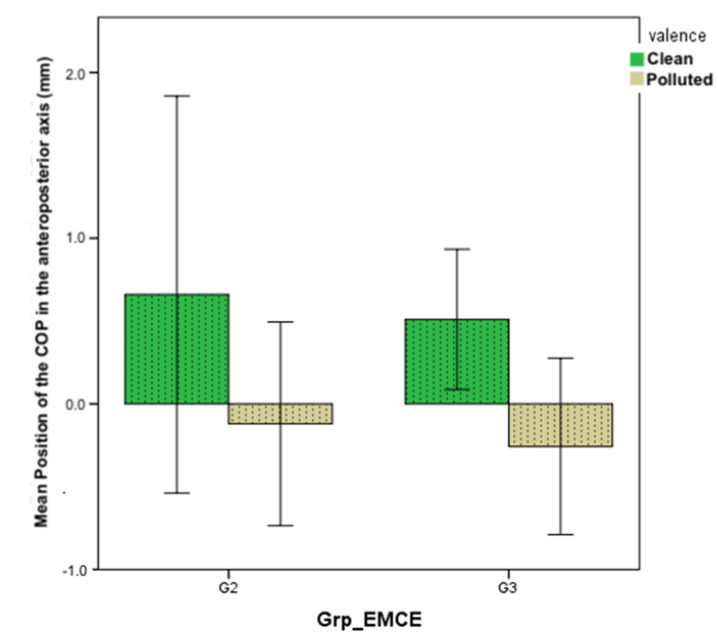
Shift of the COP in the AP axis according to the level of motivation towards ecological behaviors assessed by the WES scale.

Correlations with the COP movement averages towards “polluted” or “clean” scenes show that the more motivated participants are the more they tend to avoid a polluted landscape. However, participants with moderate or high motivation all tend to approach “clean” landscapes (Figure 8). No significant effect is shown (*p* = 0.57).

### 3.5. Correlations

The correlations between the postural and physiological data, the ratings and the measurements obtained by the scales of motivation and attachment to nature were obtained (Table 2). The subjective evaluations of the “pleasure” and “desire to approach” dimensions were correlated with the displacement of the COP-AP (*p* = 0.034 and *p* = 0.041). The physiological data were also correlated with the posturographic parameters; the galvanic response was correlated with the displacement of the COP-AP (*p* = 0.023).

## 4. Discussion

This study is included in a broader project created to study our relationships with environmental pollution and to explore whether perceiving a “polluted” landscape would induce an emotional reaction that could promote certain behavior. This project is important for understanding the incongruity between global social understanding that environmental pollution requires a behavioral change and the incapacity of long-term, significant change in individual behavior. To this aim, after having explored the subjective feelings evoked by clean or polluted environmental scenes [17], we wanted to explore the peripheral nervous side of the perception of such scenes and, more particularly, the motor, posturographic, and physiological correlates of their perception.

Our subjective ratings confirmed that polluted scenes evoked significantly higher displeasure and withdrawal. These results are in accordance with those of a previous study [17] which reported significantly higher pleasure and approach desire in response to the perception of clean scenes as compared to polluted ones. These results validate the selection of the stimuli in this preliminary study, which were kept for further studies (i.e., the present study), with the polluted stimuli inducing the highest displeasure ratings and avoidance desire ratings. The subjective ratings recorded in response to clean and polluted scenes have been extensively discussed in this study [17].

When examining the peripheral correlates recorded in response to polluted and clean environmental scenes, our study is, to our knowledge, the first to report a differential modulation of postural control regarding the degree of pollution of the depicted scenes. Interestingly, our results are in accordance with our primary hypothesis of an avoidance behavior in response to polluted scenes, in contrast to an approach behavior in response to clean scenes. As reported in Table 2, significant correlations have been found between anteroposterior displacement of the COP, and the subjective ratings of “pleasure” (*p* = 0.034), “approach” (*p* = 0.041), and electrodermal activity (*p* = 0.023). When comparing these results with those obtained in several past studies [10,11,12], it seems important to raise the following points: (i) such significant results regarding mean postural modulation in the working model of empathy for pain have been reported only recently [12], and mainly the modulation of the corresponding posturographic correlates seems smaller; and (ii) the correlation between posturographic and subjective data was only reported in one study [12], with negative correlations for the “displeasure” and “avoidance desire” dimensions, but only in a “mental simulation” condition. Therefore, our results verify the high utility of our environmental database [12] and, more particularly, of the subsample of pictures chosen for the present study to dichotomize the responses associated with perceiving clean landscapes as compared to polluted landscapes. Our results demonstrate a clear association between subjective pleasure, clean landscape perception, and approach-type behavior on one hand, and displeasure, polluted landscape perception, and avoidance-type behavior on the other hand. In that sense, our results are in perfect accordance with the biphasic theory dimension of emotions [24], which conceptualizes pleasant stimuli as inducing an approach-type behavior and unpleasant stimuli as inducing withdrawal-type behavior. The clarity of our results is in contrast to the complexity of those in the literature on the posturographic correlates of emotional information processing. Recently, Lelard et al. [7] reviewed this literature. They noted that all the considered studies demonstrated an effect of unpleasant pictures on postural control, but they also remarked on the complexity of these responses with some of them reporting withdrawal behavior and others reporting freezing responses. Through the working model of empathy for pain, our studies [10,12] even reported participants showing a clear approach-like behavior towards painful (i.e., unpleasant) stimuli, becoming a withdrawal-type behavior when they were asked to mentally simulate the scenes. Lelard et al. [7] and Horslen and Carpenter [25] support the idea that postural responses depend more on arousal than on the valence of the stimuli. As our results demonstrate a clear dichotomy between polluted and clean scenes regarding subjective ratings and therefore valence, we argue that the *complexity* of the postural responses depends on arousal and that the valence index of the stimuli seems to produce clearer responses. In our picture database validation study [17], numerous different factors appeared to influence the subjective ratings of polluted and clean scenes (gender, personal history, etc.) and we broadly discussed our results through the extensive literature explaining the importance of personal experience, personal immersion, and many other processes in the global hedonic experience produced by viewing a landscape. Here, by looking at the clarity of the present results, we conclude the utility of posturography (and, by extension, of the subsample of pictures chosen for the present study) for shedding light on powerful and regular motor tendencies between participants and for measuring a robust behavioral output, the major components of which are emotional processes induced by viewing a clean or polluted landscape. Even if the subjective emotional experience of landscape viewing encompasses complex articulations between cognitive processes, our results show that, ultimately, the posturographic indexes reflect the final production of these interactions.

Following a previous posturographic study conducted through the working model of empathy for pain [11] and our initial database validation study [17], we also hypothesized that the mental simulation effect would operate in the framework of pollution perception. However, this has not been the case, as we were unable to report any significant effect of mental simulation as compared to passive viewing for either subjective ratings or for posturographic data. In previous studies, this mental stimulation effect was at the foreground of two curious effects that we observed: (i) a dichotomy between subjective (ratings) and objective (posturographic) measures [12] with a systematically reported avoidance through painful scenes as opposed to an objective approach-type behavior in a passive viewing condition; and (ii) a modulation of the posturographic correlates towards painful scenes with a dose-dependent effect [12], where posturography demonstrated an approach-type behavior towards painful stimuli when passively viewing the stimuli, which became a withdrawal-type behavior when participants were instructed to mentally simulate the scenes. In our previous studies, we discussed this effect of mental simulation in terms of temporal deployment of conscious mechanisms under the influence of mental simulation instruction. These mechanisms involve conscious control of posture restoring an early motor response based on rather automatic processes to a later, controlled response that integrates more moral and societal aspects. Regarding our results, in the case of environmental stimulus perception, because mental simulation does not operate, this late response may be involved from the very beginning of the postural response developed towards environmental stimuli.

We analyzed the electrodermal activity relative to the maximum amplitude of the phase signal obtained for each stimulus. This is a component that varies rapidly and is considered to be an adequate measure when visual stimuli are presented. In subjects exposed to emotional stimuli, the amplitude of the electrodermal response increases linearly with the perceived excitation [26]. We observed a higher electrodermal response when viewing polluted landscapes without a significant effect (Figure 5). These images elicited an increase in the voiced electrodermal activity response, consistent with the results reported in other studies using visual stimuli [25,27] or emotional words [28]. Importantly, electrodermal activity is considered a good predictor of emotional arousal, suggesting that polluted landscapes are more exciting.

Another physiological indicator we employed was heart rate variability (HRV), which is considered as an index of motivational and emotional states [29]. We tended to observe a relatively high overall HRV when participants were shown polluted environmental scenes. Similar results have also been reported in a number of studies using emotional images as stimuli, with increased HRV linked to a feeling of disgust [30], and observed, when perceiving images of fearful faces [31] or food [32]. Our results do not show a significant effect of physiological responses, which could be explained by the lower excitatory level of the polluted landscapes than the images used in the later studies. These results could also be explained by interindividual variability among the participants (e.g., circadian rhythm, diet, medication).

To highlight some interindividual traits, we used two scales to focus on subjects’ motivational criteria and their state of attachment to nature. The first scale assesses the concept of “inclusion of nature in the self” which refers to the consideration of nature as part of oneself. It measures the implicit connection that individuals perceive between themselves and nature (INS; [18,33]). Our results show that the more strongly one is attached to nature, the more one reacts emotionally towards polluted landscapes, without a significant effect. This emotional response may play a role in pro-environmental decision making and action [34]. Higher scores correlate with a movement tendency toward clean landscapes and an avoidance tendency toward polluted landscapes. To explore the motivational profile of participants, we used the EMCE scale to conclude that those with high motivational scores showed avoidance behavior towards polluted scenes. Note that the limitation of using these two scales is related to the relatively small sample size, which results in smaller subgroups and prevents a significant effect from being concluded. Overall, this study confirms that the experimental paradigm used is reliable for the study of emotional response during the perception of environmental landscapes

## 5. Conclusions

Our results demonstrate the merit of using posturography to study the modulation of the postural attitude in terms of the degree of environmental pollution. The main contribution of these results can be understood through the comparison of the present results and those obtained in our previous research focusing on the postural correlates of painful vs. non-painful stimulus presentation. First, it is important to consider that polluted scenes nonetheless convey an emotional component as well as a “societal” or “ethical” component, which can be considered absent or at least far less important in painful stimuli. In that sense, it is important to note that the postural correlates of painful stimulus perception on one hand and of polluted landscapes on the other hand differ in different aspects: (i) the dichotomy between subjective and objective measures reported in the framework of painful stimulus perception, which is not present in the present study; and (ii) the nature of the early posturographic reaction, which differs when exposed to painful stimuli and to polluted landscapes. These differential aspects have been at the foreground of our interpretations of the present data regarding the nature of the cognitive processes involved and the postural attitude developed toward either polluted or clean landscapes. In that sense, we demonstrated that the mental simulation effect, which could be an important lever in the functional context of pain perception, does not strongly operate in the functional context of pollution perception. Thus, beyond specifying the nature of the cognitive processes at work and influencing postural reactions in pollution and hoping to find new levers to influence them (by measuring the modulation of postural reactions by these levers), it would be interesting to conduct new studies using new means of visual stimulation (such as virtual reality devices, for example) to explore in greater detail the effect of immersion of subjects on their postural reactions in response to pollution.

## Figures and Tables

**Table 1:** The means ± standard deviation of the different parameters derived from the heart rate variability for each condition. RMSSD = Root Mean Square of Successive R-R intervals; SDNN = Standard Deviation of all NN intervals.

	“Passive Observation”		“Mental Simulation Observation”	
	Clean	Polluted	Clean	Polluted
**RMSSD (ms)**	38.31 ± 14.32	44.48 ± 19.98	44.00 ± 16.59	45.84 ± 26.56
**SDNN (ms)**	35.37 ± 13.63	41.90 ± 19.06	40.16 ± 14.22	42.59 ± 19.21

**Table 2:** Correlational table between the posturographic, subjective, physiological and psychometric data.

	Pleasure	Approach	EDA	HRV	INS	Motivation
**COP-AP**	0.034 *	0.041 *	0.02 *	0.683	0.971	0.765
**Pleasure**		7.1 × 10^−66^ *	0.639	0.419	0.558	0.605
**Approach**			0.578	0.457	0.335	0.385
**EDA**				0.141	0.035 *	0.348
**HRV**					0.562	0.02 **
**INS**						0.022 *

Acronyms: COP-AP = Center of Pressure-AnteroPosterior displacement; HRV = Heart Rate Variability; EDA = electrodermal activity; INS = *Environmental Concerns (INS;* Schultz, 2001) scale. Significant differences are indicated as: * *p* < 0.05; ** *p* < 0.01.

## Data Availability

The data presented in this study are available on request from the corresponding author.
